# New insights of altered lipid profile in Fragile X Syndrome

**DOI:** 10.1371/journal.pone.0174301

**Published:** 2017-03-23

**Authors:** Artuela Çaku, Nabil G. Seidah, Audrey Lortie, Nancy Gagné, Patrice Perron, Jean Dubé, Francois Corbin

**Affiliations:** 1 Department of Biochemistry, Université de Sherbrooke, Sherbrooke, Québec, Canada; 2 Nabil G. Seidah, Laboratory of Biochemical Neuroendocrinology, Clinical Research Institute, affiliated to the Université de Montréal, Montréal, Québec, Canada; 3 Department of Paediatrics, Université de Sherbrooke, Sherbrooke, Québec, Canada; 4 Department of Medicine, Université de Sherbrooke, Sherbrooke, Québec, Canada; Centre National de la Recherche Scientifique, FRANCE

## Abstract

**Background:**

Fragile X Syndrome (FXS) is the main genetic cause of autism and intellectual deficiency resulting the absence of the Fragile X Mental Retardation Protein (FMRP). Clinical picture is characterized by cognitive impairment associated with a broad spectrum of psychiatric comorbidities including autism spectrum disorders and attention-deficit/hyperactivity disorders. Some of these disorders have been associated with lipid abnormalities and lower cholesterol levels. Since lipids are important for neuronal development, we aim to investigate the lipid profile of French Canadian-FXS individuals and to identify the altered components of cholesterol metabolism as well as their association with clinical profile.

**Methods:**

Anthropometric data were collected from 25 FXS individuals and 26 controls. Lipid assessment included: total cholesterol (TC), triglycerides, LDL, HDL, ApoB, ApoA1, PCSK9, Lp(a) and lipoprotein electrophoresis. Aberrant and adaptive behaviour of affected individuals was respectively assessed by the ABC-C and ABAS questionnaires.

**Results:**

FXS participants had a higher body mass index as compared to controls while 38% of them had TC<10^th^ percentile. Lower levels of LDL, HDL and apoA1 were observed in FXS group as compared to controls. However, PCSK9 levels did not differ between the two groups. As expected, PCSK9 levels correlated with total cholesterol (r_s_ = 0.61, *p = 0*.*001*) and LDL (r_s_ = 0.46, *p = 0*.*014*) in the control group, while no association was present in the FXS group. An inverse relationship was observed between total cholesterol and aberrant behaviour as determined by ABC-C total score.

**Conclusion:**

Our results showed the presence of hypocholesterolemia in French Canadian-FXS population, a condition that seems to influence their clinical phenotype. We identified for the first time a potential underlying alteration of PCSK9 function in FXS that could result from the absence of FMRP. Further investigations are warranted to better understand the association between cholesterol metabolism, PCSK9, FMRP and clinical profile.

## Introduction

Fragile X Syndrome (FXS) is a genetic condition caused by an expansion mutation of a CGG repeat sequence in the *FMR1* gene resulting in the methylation of the promoter; the silencing of the gene leads to a failure to express the fragile X mental retardation protein (FMRP) [1, 2]. FMRP is an ubiquitous protein, mostly expressed in neurons where it regulates the postsynaptic protein synthesis required for synaptic plasticity [3]. The lack of this protein leads to an alteration of cognitive functions and hence to a typical phenotype characterized by varying degrees of intellectual disability (ID). The FXS individuals also present anatomical abnormalities associated with a vast variety of medical conditions including neurologic, cardiac, gastrointestinal, ophthalmologic and/ or ear-nose-throat problems [4].

Metabolic anomalies have been recently described in FXS population. More specifically, low plasma cholesterol levels have been first reported in a retrospective study of 103 FXS subjects. Basic components of a non-fasting lipid profile including total cholesterol, low-density lipoprotein cholesterol (LDL) and high density lipoprotein cholesterol (HDL) where all significantly reduced in FXS males as compared to age and gender-matched American reference population [5]. Low HDL levels have been as well reported in another prospective study of 23 Polish males with FXS in which fasting lipid profile was compared to an age matched group of 24 controls [6]. However, to the best of our knowledge no study has tried to identify the underlying factors related to hypocholesterolemia.

Cholesterol is an essential structural and functional cellular component. It is mainly synthesized in the liver and circulates in plasma within lipoproteins. The latter are plasma particles that contain in addition to cholesterol phospholipids, triglycerides and proteins (called apolipoproteins) [7]. Their major function is the transport of cholesterol and fatty acids between liver and peripheral tissues. There are five mains lipoproteins, differing in the relative proportion of cholesterol/triglycerides and the type of apolipoprotein: chylomicrons (transport diet triglycerides from intestine to tissues), VLDL (contain apolipoprotein B-100 [ApoB] and transport triglycerides from tissues to liver), LDL (contain ApoB and transport cholesterol from liver to tissues), HDL (contain apolipoprotein A1 [ApoA1] and transport cholesterol from tissues back to liver) and Lp(a) (one of the five principal plasma lipoproteins, its levels concentration are genetically determined and do not vary with age or gender) [8]. Cholesterol uptake is mediated by LDL receptors (LDLR), which are located on the cell surface and are regulated by PCSK9 (proprotein convertase subtilisin/kexintype 9). PCSK9 plays an important role in cholesterol homeostasis. Once it binds to the LDLR, it induces receptor degradation [9]. Thus, low PCSK9 levels increase LDLR expression on cell surface resulting in increased clearance of LDL, which leads to hypocholesterolemia.

We therefore sought to investigate a complete panel of lipid components including all the above-mentioned lipoproteins as well as PCSK9 levels. The aims of this study were to investigate first whether cholesterol levels are decreased in our population of French-Canadian FXS subjects, and then to identify underlying factors related to low cholesterol levels.

## Methods

### Study population

The study population included two groups: an FXS group of 26 affected individuals and a control group of 25 healthy individuals. The former was recruited through the Fragile X Clinic, at *CIUSS de l’Estrie-CHUS*, Quebec, Canada and the inclusion criteria comprised (1) male or female; (2) aged from 12 to 50 years old; (3) having a confirmed diagnosis of FXS by southern blot and polymerase chain reaction; (4) availability of parent/caregiver for the clinic visit and cognitive assessments. The control group was recruited from the community and the inclusion criteria comprised (1) male or female; (2) aged from 18 to 50 years old; (3) having at least a high school diploma or its equivalent. Participants in each group were excluded if they were treated with lipid lowering drugs or suffered from malignancy, liver disease, critical illness or malabsorption / malnutrition. The study was conducted between January and June 2015 and was approved by the Scientific and Ethical Board of the Research Center of *Centre Hospitalier Universitaire de Sherbrooke (CHUS)* [14–220]. Written, informed consent was obtained from each participant of the control group. Written consent was also obtained from the caregiver/guardian on behalf of the mentally disabled minors and adults with FXS.

### Data collection

Anthropometric data including, height, weight, waist, body mass index (BMI) and blood pressure were measured. Blood samples were collected from all participants after a period of 8–12 hours fast. Total cholesterol, triglycerides and HDL-C were measured by enzymatic methods (Modular Roche P800), while LDL-C was calculated using the Friedewald formula. ApoB, ApoA1 and Lp(a), were measured by immunoturbidimetric assays (Roche Diagnostics, Cobas 501 analyser). All these tests were performed at the core laboratory of *CIUSSS de l’Estrie*-*CHUS*. Otherwise, PCSK9 was determined by ELISA at the Laboratory of Biochemical Neuroendocrinology, Clinic Research Institute of Montreal [10] while lipoprotein electrophoresis was performed at the Lipid Research Center of the *Centre Hospitalier de l’Université Laval*. Furthermore, family history of cardiovascular disease (CVD), comorbidities and medications that participants were taking at the time of blood sample collection were recorded.

Clinical phenotype of FXS subjects was assessed by two questionnaires that were completed by their parents. The FXS version of Aberrant Behavioural Checklist—Community (FXS-ABC) and the Adaptive Behaviour Assessment System® Second Edition (ABAS-II) have been previously validated in FXS population and used in clinical trials [11]. More precisely, the FXS-ABC is a 58-item rating scale where each item has a score from 0 (not at all a problem) to 3 (problem is severe in degree) and the total score ranges from 0 to 174. It evaluates six domains of maladaptive behaviour including: Hyperactivity, Social Avoidance, Socially Unresponsive/Lethargic, Stereotypy, Irritability and Inappropriate Speech [12]. The Adaptive Behavior Assessment System® Second Edition (ABAS-II) provides a complete assessment for three areas of adaptive behavior (conceptual, practical and social). Each item has a score from 0 (not able to perform the task) to 1 (never or almost never performs the task), 2 (performs the task sometimes), or 3 (always or almost always performs the task). Raw scores were calculated by tabulating totals from the items and then converted into composite scores [13].

### Statistical analyses

Data distributions are reported as mean ± standard deviation for continuous variables and as number (percentage) for categorical variables. Statistical differences between groups were tested using student’s t-test or Wilcoxon rank sum test. Lipid parameters (including total cholesterol, triglycerides, HDL-C and LDL-C) were analysed by gender. Profiles of the male FXS participants were compared to the age-matched data from the Canadian Heart Health Survey (1986–1992) population study [14]. Furthermore, ApoB and ApoA1 for each male participant were compared to age and gender-based normative data from Canadian Heart Health Surveys Research Group (1989–1990). The former study included lipid fasting results of 18,555 men and women from 10 provinces of Canada while the latter study included stratified fasting samples of 1755 men and 1764 women selected from the provinces of Saskatchewan and Quebec. Box-and-Whisker plots were used to show the distribution of lipid components (for each group) while statistical differences were calculated using one simple Wilcoxon signed-rank tests (difference between FXS and age matched population). For FXS group, lipid levels were categorized as being below the 5^th^ and the 10^th^ percentile for age group of normative data. The difference of proportions was tested using Fisher’s exact test. Spearman’s correlation coefficient was used to assess the association of lipid components with anthropometric data, FXS-ABC and ABAS-II (domain and total) scores. Multiple linear regression analyses adjusting for age and sex were used to test the impact of the anthropometric data on lipid profile. For all statistical tests, p-value<0.05 was considered significant. All statistical analyses were performed using R (version 3.0.1).

## Results

### Population characteristics

Participant’s characteristics for FXS and control group are presented in Table 1. As expected in the FXS group the majority of participants were males (88%). Significant statistical differences were observed between two groups with FXS subjects having higher BMI (*p = 0*.*005*) and higher rate of family history of CVD (*p = 0*.*029*) as compared to control group. Furthermore, more than 50% of FXS individuals were treated with at least one psychoactive medication.

**Table 1:** Population characteristics.

		FXS group	Control group	*p*-value
		(n = 26)	(n = 25)	
**Age (mean ± SD)**		25.9 ± 6.9	24.3 ± 5.8	*ns*^*a*^
**Gender (males)**		23 (88.5%)	14 (56%)	*0*.*012*^*b*^
**Ethnicity**				
	Caucasians	26 (100%)	23 (92%)	
	Asian	0 (0%)	1 (4%)	*ns*^*b*^
	Other	0 (0%)	1 (1%)	
**Waist cm (mean ± SD)**				
	Male	91.39 ± 16.88	83.71 ± 10.62	*ns*^*c*^
	Female	102.33 ± 15.95	74.55 ± 7.39	*0*.*013*^*c*^
**BMI kg/m**^**2**^ **(mean ± SD)**				
**Male**		26.0 ± 5.9	23.5 ± 3.9	*ns*^*c*^
**Female**		33.5 ± 7.8	21.5 ± 1.7	*0*.*005*^*c*^
**Family history of CVD N**		11 (42.3%)	3(12.0%)	*0*.*029*^*b*^
**FXS diagnosis (males)**				
Full mutation		19		
Mosaic		4		
**CGG pb (range in mutated males)**		600–2000		
**Comorbidities (N)**				
	Neurologic	3		
	Endocrine	2		
	Metabolic	1		
	Autism	1		
	ADHD	4		
**Psychoactive medication (N)**				
	≥2	10		
	1	4		
	None	12		
**Class of psychoactive medication**				
	Antipsychotics	5		
	SSRIs	8		
	Stimulants	5		

N:number.

^a^ t.test for unpaired groups.

^b^Fisher's exact test.

^c^Mann Whitney test.

### Lipid profile

**Fig 1:**
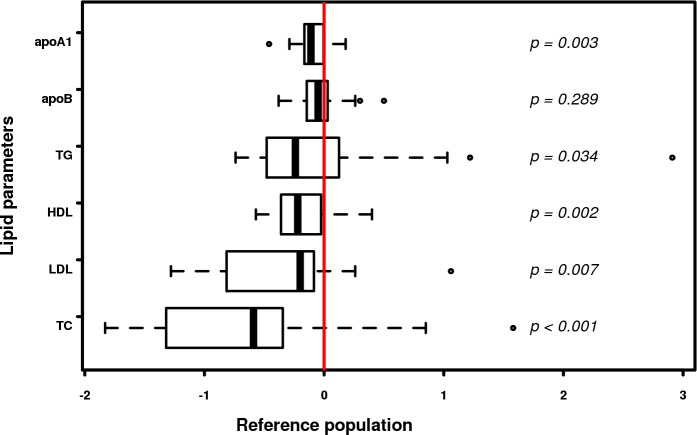
Lipid parameters in FXS males. Distribution of lipid components are illustrated with box and whisker plots, with the bottom and top of the box representing first and third quartiles and the band inside the box the median (the bottom whisker extends to the smallest corresponding concentration from first quartile and top whisker extends to the largest corresponding concentration from third quartile). Lipid components are compared to corresponding reported levels in normal population, matched for age and sex (red line) and p-value for difference is shown for each lipid component. TC: total cholesterol; TG: triglycerides; apoB: apolipoproteinB100; apoA1: apolipoproteinA1.

Considering the limited number of female FXS participants (n = 3), analysis of lipid profiles was not pursued in this group. Male FXS participants had statistically significant lower levels of all lipid parameters as compared to the normal male population (adjusted for age regarding total cholesterol (*p<0*.*001*), HDL-C (*p = 0*.*002*), triglycerides (*p = 0*.*034*) and LDL-C (*p = 0*.*007*) (Fig 1). Low plasma levels of ApoA1 (*p = 0*.*003*) were also confirmed by lipoprotein electrophoresis where 46% of FXS individuals had a decrease of alpha-lipoproteins. In addition, up to 30% of FXS subjects had very low cholesterol levels (<5^th^ centile), while 27% to 42% of this group had total cholesterol, HDL-C and LDL-C levels below the 10^th^ centile relative to the normative population (Table 2). Otherwise, there were no significant statistical differences between the control group and the normative population (adjusted for age and sex), regarding total cholesterol (*p = 0*.*231*) and triglycerides (*p = 0*.*435*). The control group was mainly composed of young students with a normal BMI and a high level of physical activity. Therefore their HDL-C and LDL-C levels were respectively significantly higher (*p* = 0.008) and lower (*p* = 0.002) as compared to the reference group.

**Table 2:** Fraction of FXS individuals below the 5^th^ and 10^th^ centile of normative data.

	< 5^%-ile^		< 10^%-ile^	
Lipid parameters	Fraction N (%)	*p*-value^a^	Fraction N (%)	*p*-value^a^
**Total cholesterol**	6 (23)	0.002	9 (35)	<0.001
**HDL**	8 (31)	<0.001	11 (42)	<0.001
**LDL**	5 (19)	0.009	7 (27)	0.01
**Triglycerides**	5 (19)	0.009	7 (27)	0.01
**ApoB**	3 (12)	ns	6 (23)	0.041
**ApoA1**	2 (8)	ns	4 (15)	ns

^a^Fisher’s exact test.

**Fig 2:**
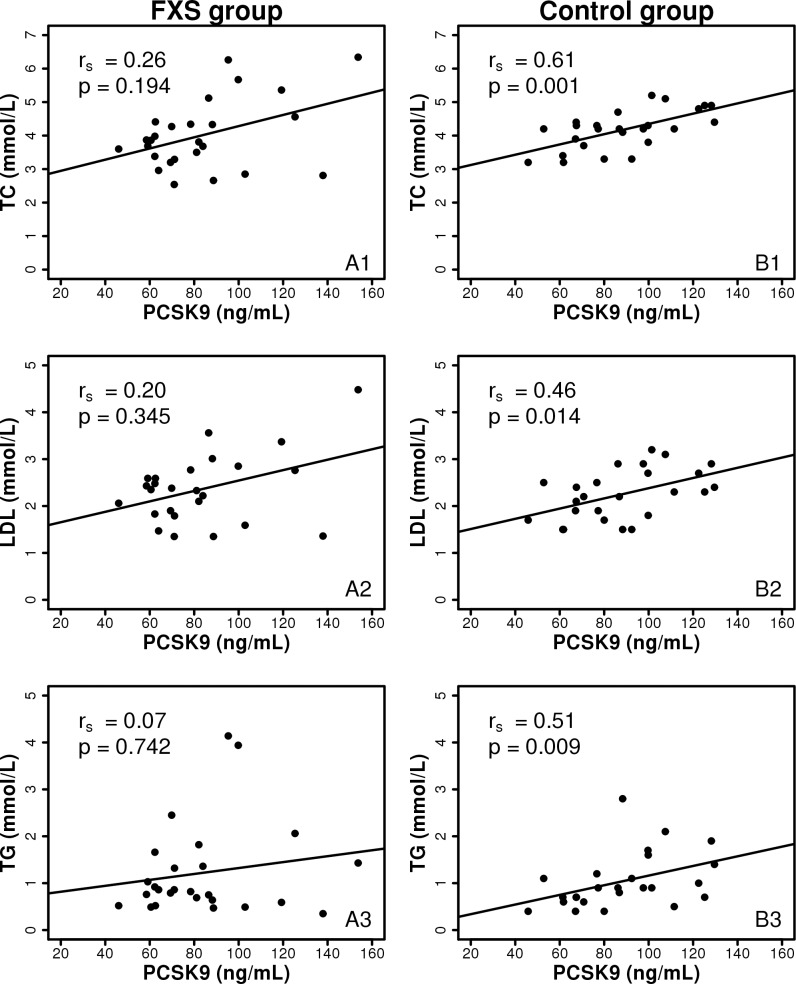
Correlation between PCSK9 levels and lipid parameters. Spearman correlations: between PCSK9 and total cholesterol in FXS group (A1) and control group (B1); between PCSK9 and LDL in FXS group (A2) and control group (B2); between PCSK9 and triglycerides in FXS group (A3) and control group (B3). TC: total cholesterol; TG: triglycerides.

No significant difference was observed between FXS and control groups regarding PCSK9 plasma concentrations. As expected, PCSK9 levels correlated with total cholesterol (r_s_ = 0.61, *p =* 0.001), LDL-C (r_s_ = 0.46, *p =* 0.014) and triglycerides (r_s_ = 0.51, *p =* 0.009) in control group. However, this association was lost in the FXS group (Fig 2). The results were consistent for FXS male cohort as well. Furthermore Lp(a) levels did not differ between the two groups.

### Association of lipid parameters with anthropometric data

For FXS males, there was a significant association between BMI and triglycerides (Spearman’s rho = 0.68, *p* < 0.001) and HDL-C (Spearman’s rho = -0.59, *p* = 0.003). Similarly, these associations were significant in the FXS group as a whole including both males and females (for triglycerides Spearman’s rho = 0.77, *p* < 0.001 and for HDL-C Spearman’s rho = -0.53, *p* = 0.005). Multiple linear analyses while adjusting for age and sex confirmed a statistically significant association between BMI and both triglycerides (estimate [95% CI] = 0.08 [0.02–5.46], *p* = 1.7 x 10^−5^) and HDL-C (estimate [95% CI] = -0.02 [0.01 –-3.69], *p* = 0.001). There was a mild increase of 0.08 mmol/L in triglycerides and a 0.02 mmol/L decline in HDL per each unit increase in BMI. Total cholesterol and LDL were not associated with BMI even before adjustment for age and sex.

### Association of lipid parameters with clinical profile

ApoB and ApoA1 measurements were performed along with LDL and HDL to estimate the beta and alpha lipoproteins levels. Since their levels are less affected by diet, we used them to assess the relationship between lipid parameters and clinical profile. As shown on Table 3, negative relationships were observed between several lipid parameters and ABC-C’s total or subdomains scores. ApoB showed a statistically significant association with total score (r_s_ = -0.41, *p* = 0.044) in addition to hyperactivity (r_s_ = -0.56, *p* = 0.005), stereotypy (r_s_ = -0.46, *p* = 0.024) and language (r_s_ = -0.47, *p* = 0.020). When ABAS domain scores were correlated with lipid parameters, positive associations reached significance for practical and conceptual domain with apoB (respectively r_s_ = 0.57, *p =* 0.004 and r_s_ = 0.41, *p =* 0.049) and apoA1 (respectively r_s_ = 0.52, *p =* 0.009 and r_s_ = 0.57, *p =* 0.004). Lp(a) levels did not differ between two groups. However, consistent associations were also observed between Lp(a) levels and conceptual domain (r_s_ = 0.53, *p* = 0.036), practical domain (r_s_ = 0.57, *p* = 0.004) as well as ABAS global score *(*r_s_ = 0.51, *p* = 0.013).

**Table 3:** Association of lipid parameters with cognitive function.

Questionnaire	Total cholesterol		ApoB		ApoA1		Lp(a)	
	r_s_	p	r_s_	p	r_s_	p	r_s_	p
**ABC-C**								
Hyperactivity	**-0.48**	**0.017**	**-0.56**	**0.005**	-0.23	0.238	-0.11	0.607
Social avoidance	-0.24	0.261	-0.33	0.119	-0.23	0.274	-0.32	0.133
Unresponsiveness	-0.31	0.148	-0.38	0.067	**-0.43**	**0.036**	-0.34	0.104
Stereotypy	-0.38	0.071	**-0.46**	**0.024**	-0.37	0.076	-0.28	0.185
Irritability	-0.22	0.308	-0.28	0.191	-0.12	0.584	-0.29	0.171
Language	**-0.43**	**0.035**	**-0.47**	**0.020**	**-0.49**	**0.016**	-0.36	0.086
Total score	-0.34	0.105	**-0.41**	**0.044**	-0.34	0.107	-0.29	0.164
**ABAS**								
Conceptual	0.38	0.067	**0.41**	**0.049**	**0.57**	**0.004**	**0.53**	**0.036**
Practical	**0.49**	**0.016**	**0.57**	**0.004**	**0.52**	**0.009**	**0.57**	**0.004**
Social	0.26	0.223	0.39	0.059	0.27	0.192	0.40	0.055
Total score	0.32	0.133	0.39	0.070	**0.51**	**0.014**	**0.51**	**0.013**

ABC: Aberrant Behavioural Checklist—Community; ABAS-II Adaptive Behaviour Assessment System® Second Edition (ABAS-II).

## Discussion

In this cohort of French Canadian individuals with SXF, total cholesterol originating from both alpha and beta lipoproteins was lower as compared to the reference French Canadian population adjusted for age and sex. Results suggest that males across all ages are affected. However results in female are not conclusive considering the small sample size.

These findings confirmed the previous reported results from a retrospective study where non-fasting lipid profile data were collected from 103 FXS North Americans (mainly were of Caucasian ethnic group) [5]. Their results showed a decrease of total cholesterol, LDL and HDL in FXS males as compared to an NHANES study population. In the present study we measured ApoB and ApoA1 in addition to a fasting lipid profile to better define the presence of low levels of both alpha and beta lipoproteins. In the present study 38% had total cholesterol levels below 10^th^ percentiles whereas the sub-mentioned study showed 27% of FXS participants falling in this category. Fasting state and differences in the normative data of reference populations may account for between-study variations. Another recent study done in 23 Polish with FXS showed only a significant decrease of HDL-C [6]. However, their lipid levels were compared to a small sample size of 24 healthy males. Moreover, the authors recognised their study was not powered to detect a significant difference in TC and LDL-C. Our sample size was also small; nevertheless to bypass this limitation we compared the lipid parameters levels of each group with their reference population after adjustment for age and sex.

Several risk factors should be considered when evaluating a lipid profile. First, genetic predisposition is an important risk factor depending on the ethnic origin. Indeed, French Canadians have a higher frequency of familial hypercholesterolemia (1/270) as compared to other Western countries (1/500) [15]. Second, psychoactive medications including antipsychotics [16] and/or SSRI drugs [17] can increase triglycerides, LDL-C and decrease HDL-C. Third, uncontrolled diet (frequent in FXS population) associated with high BMI is an additional factor that contributes to high triglyceride ± low HDL-C levels [16]. Regardless of the presence of all these risk factors, FXS males had lower levels of total cholesterol, triglycerides and LDL-C. These results suggest that the absence of FMRP could be a determinant factor of circulating cholesterol levels.

Hence, this study aimed to identify the intermediate factors implicated in cholesterol metabolism that are altered in FXS and PCSK9 is one marker that plays an important role in cholesterol homeostasis. PCSK9 is secreted into the plasma by the liver, it binds to LDL-C receptor at the surface of hepatocytes, thereby preventing its recycling and enhancing its intracellular degradation, resulting in reduced LDL-cholesterol clearance [9]. In addition, VLDL receptors have been confirmed as well as PCSK9 target proteins and positive associations have been shown between PCSK9 and triglyceride plasma levels [18]. Our results suggest an alteration of PCSK9 function through the presence of: (1) no significant difference between FXS and control group regarding PCSK9 levels; (2) lack of correlation between PCSK9 and total cholesterol, LDL-C and triglycerides in FXS individuals, while a statistically significant correlation was shown as expected in the control group. Moreover, statins are lipid lowering drugs that not only inhibit HMG-CoA enzyme but in the same time increase PCSK9 levels [19]. We have previously reported in FXS subjects a significant decrease of LDL-C (36%) and triglycerides (27%) under lovastatin treatment [20], which is more than expected; lovastatin 40 mg is known to decrease triglycerides up to 14% and LDL-C up to 25% [21]. This over-response to lovastatin observed in FXS individuals suggests that lipid lowering effect of statins is probably not counterbalanced by the associated increase of PCSK9 expression. Thus PCSK9 is normally expressed in FXS individuals; however it does not function properly. An abnormal expressed protein in the context of FMRP deficiency could alter binding sites, escort or degradation of the PCSK9-LDLR complex [9, 18]. Additional *in-vitro* studies are warranted to further explore the underlying mechanism of altered PCSK9 function and identify potential biomarkers that could monitor the efficacy of new drugs for FXS treatment.

Questionnaires evaluating the aberrant behaviour (ABC-C) or the adaptive function (ABAS) have been widely used to assess clinical profile of FXS subjects. Negative relationships were observed between total cholesterol, ApoB, ApoA and ABC-C domains while positive associations were observed between these parameters and ABAS domains. These results suggest low cholesterol levels could exacerbate the aberrant behavior and decline the adaptive function.

The results of the present study should be interpreted in light of a few limitations including the small sample size and the lack of age and sex matching between two groups. However lipid parameter levels of each group were compared with their corresponding reference population, which better supports our findings regarding low cholesterol levels in French Canadian population with FXS. Another limitation of this study is the lack of FMRP measurement. Determining FMRP in both groups could have helped us to better explore the direct effect of this protein in lipid profile; this should be considered for future studies.

In conclusion, peripheral cholesterol metabolism seems to be affected in FXS, however studies are needed to explore if brain cholesterol metabolism is also affected. Identifying the FMRP regulated mRNAs or proteins as causal factors for hypocholesterolemia could have implications not only for identifying potential FXS biomarkers but also in finding novel PCSK9 inhibitors for the treatment of hypercholesterolemia.
